# Supplementary material for: Immunoregulatory role of the gut microbiota in inflammatory depression
Source: Nat Commun. 2024 Apr 8;15:3003. doi: 10.1038/s41467-024-47273-w (PMC11001948; doi:10.1038/s41467-024-47273-w)
Supplement: Supplementary file 1 — Supplementary Information [file 41467_2024_47273_MOESM1_ESM.pdf]

Supplemental material and methods:

Clinical study

Table- S1. Clinical characteristics, inflammatory parameters and SCFAs in MDD and HCs

Variable	MDD (n=6)	HCs (n=6)	t/x^2	*P*
Gender(Male/ Female)	3/3	4/2	0.345	0.557[a]
Age (years)	26.33±7.34	26.33±8.69	0.001	1.00[b]
Education years	11.83±3.97	15.83±0.75	2.424	0.036[b*]
BMI	21.83±4.24	23.34±1.60	0.814	0.435 [b]
HAMD-17	20.33±3.67	2.50±1.38	11.143	0.001 [b*]
hs-CRP (ng/ml)	114.60±22.82	90.44±13.04	2.251	0.048[b*]
Acetic acid (μg/mL)	4.85±0.86	5.72±0.71	1.920	0.084[b]
Propionic acid (μg/mL)	0.49±0.26	0.52±0.34	0.158	0.878[b]
Butyric acid (μg/mL)	0.014±0.023	0.085±0.047	3.306	0.008[b*]
Isobutyric acid (μg/mL)	0.020±0.017	0.020±0.022	0.208	0.840[b]
Isovaleric acid (μg/mL)	0.18±0.15	0.19±0.20	0.075	0.941[b]
Valeric acid (μg/mL)	0	0.007±0.017	1.000	0.341[b]
Caproic.acid (μg/mL)	0.057±0.071	0.028±0.032	0.886	0.402[b]

All statistical tests are two-sided.

a *P* value for chi-square test.

b *P* values for two-sample t-test.

* significant difference.

Note: SCFAs, short chain fatty acids; MDD, major depressive disorder; HC, health control; BMI, Body Mass Index; HAMD, Hamilton's Depression Scale; hs-CRP, high-sensitivity C-reactive protein, IL-1β, interleukin 1β; IL-6, interleukin 6; IL-10, interleukin 10; TNF-α, tumor necrosis factor α.

Table- S2. Inflammatory parameters and permeability biomakers of intestinal mucosa in MDD and HCs

Variable	MDD (n=6)	HC (n=6)	t/x^2	*P*
TLR-4（ng/ml)	322.29±52.47	214.22±45.13	3.825	0.003 [b*]
NF-κB（ng/ml)	1254.79±120.31	884.35±105.43	6.285	0.001 [b*]
NLRP3（pg/ml)	11415.16±1710.06	5053.22±3167.30	4.329	0.001 [b*]
Caspase-1 (pg/ml)	1441.21±198.82	1210.07±260.50	1.728	0.115 [b]
Claudin-1（pg/ml)	11509.99±3780.08	17425.45±2118.70	3.344	0.007 [b*]
ZO-1（ng/ml)	2675.49±563.13	3897.93±533.04	3.862	0.003[b*]
Occludin（ng/ml)	9028.08±1649.50	12689.84±2820.90	2.745	0.021[b*]

All statistical tests are two-sided.

b *P* values for two-sample t-test.

* significant difference.

Table- S3. Clinical characteristics, inflammatory parameters and SCFAs in Inflammatory depression and Non-inflammatory depression

Variable	Inflammatory depression (n=42)	Non-inflammatory depression (n=43)	t/x^2	*P*
Gender(Male/ Female)	21/21	18/25	0.567	0.451[a]
Age (years)	22.29±6.54	26.30±10.21	2.154	0.034[b*]
Education years	12.74±3.15	13.05±3.43	0.432	0.667[b]
BMI	21.77±3.82	21.74±3.29	0.040	0.969[b]
HAMD-17	27.24±6.40	25.40±5.41	1.435	0.155[b]
HAMA	19.45±6.31	18.00±5.30	0.937	0.353[b]
hs-CRP (ng/ml)	136.21±10.22	94.54±12.50	16.809	0.001[b*]
IL-1β (pg/ml)	191.90±39.93	188.91±35.29	0.364	0.717[b]
IL-6 (pg/ml)	112.41±26.83	112.33±24.46	0.014	0.989[b]
IL-10 (pg/ml)	160.01±31.02	160.72±27.48	0.111	0.912[b]
TNF-α (pg/ml)	493.01±105.34	477.98±108.63	0.643	0.522[b]
Acetic acid (μg/mL)	3.26±2.52	2.44±2.23	1.489	0.141[b]
Propionic acid (μg/mL)	0.24±0.27	0.22±0.22	0.332	0.741[b]
Butyric acid (μg/mL)	0.037±0.056	0.024±0.030	1.286	0.202[b]
Isobutyric acid (μg/mL)	0.017±0.021	0.011±0.018	1.293	0.200[b]
Isovaleric acid (μg/mL)	0.086±0.121	0.060±0.085	1.070	0.288[b]
Valeric acid (μg/mL)	0.010±0.042	0.003±0.001	1.367	0.176[b]
Caproic.acid (μg/mL)	0.026±0.038	0.011±0.026	2.068	0.042[b*]

All statistical tests are two-sided.

a *P* value for chi-square test.

b *P* values for two-sample t-test.

* significant difference.

Note: SCFAs, short chain fatty acids; MDD, major depressive disorder; HC, health control; BMI, Body Mass Index; HAMD, Hamilton's Depression Scale; hs-CRP, high-sensitivity C-reactive protein, IL-1β, interleukin 1β; IL-6, interleukin 6; IL-10, interleukin 10; TNF-α, tumor necrosis factor α.

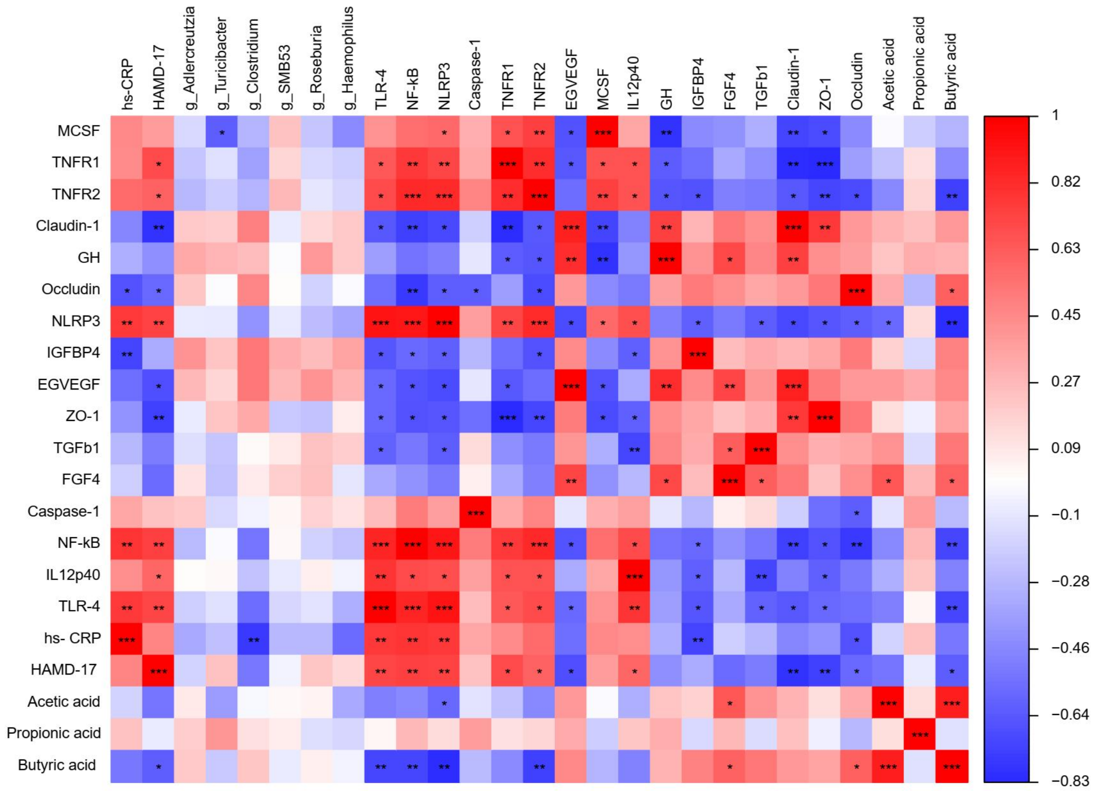

Fig-S1. The correlation analysis were performed and found the relative abundance of Clostridium were negatively correlated with the level of MCSF and hs-CRP; The inflammatory factor were negatively correlated with the permeability biomarkers (Claudin-1, ZO-1, Occludin) and SCFAs (Acetic acid, Propionic acid, Butyric acid); and positively correlated with the total score of HAMD-17. The Butyric acid was positively correlated with Occludin and negatively correlated with TLR-4, NF-κB, NLRP3, TNFR2 and HAMD-17. The Claudin-1 was positively correlated with intestinal mucosal repair markers such as GH and EG-VEGF. Pearson correlation analyses were implemented with FDR correction. The red and blue of the table represent positive and negative correlations respectively. *P*-value is marked as follows: ***: $P\leq 0.001$; **: $P\leq 0.01$; *: $P\leq 0.05$. Source data are provided as a Source Data File.

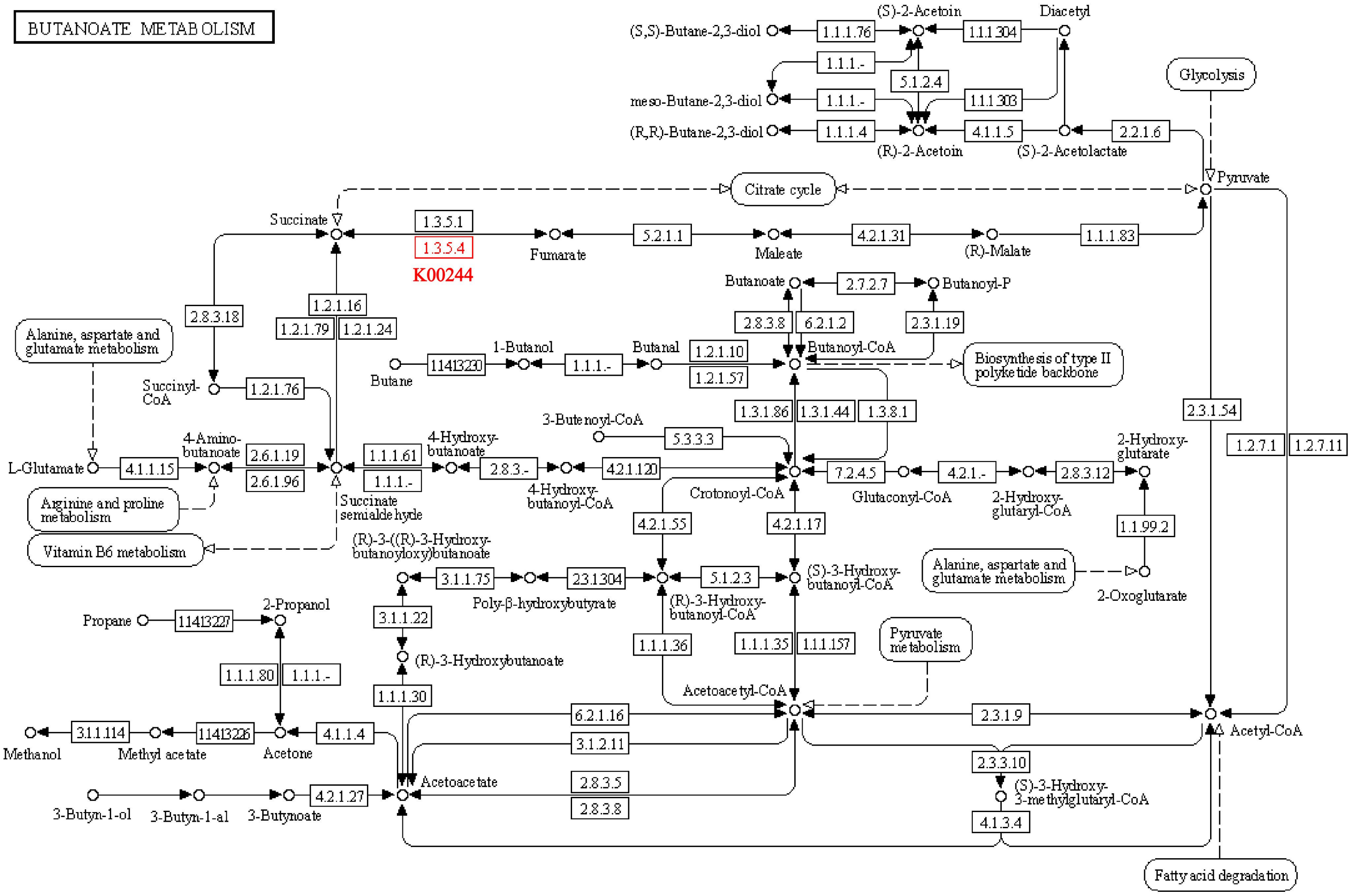

Fig- S2. butanoate metabolism (map00650) was abnormal in inflammatory depression. The different Kyoto Encyclopedia of Genes and Genomes (KEGG) orthology (KO) were searched by analyze all functional gene sequences and found the expression of K00244 (fumarate reductase A) was significantly decreased in inflammatory depression.

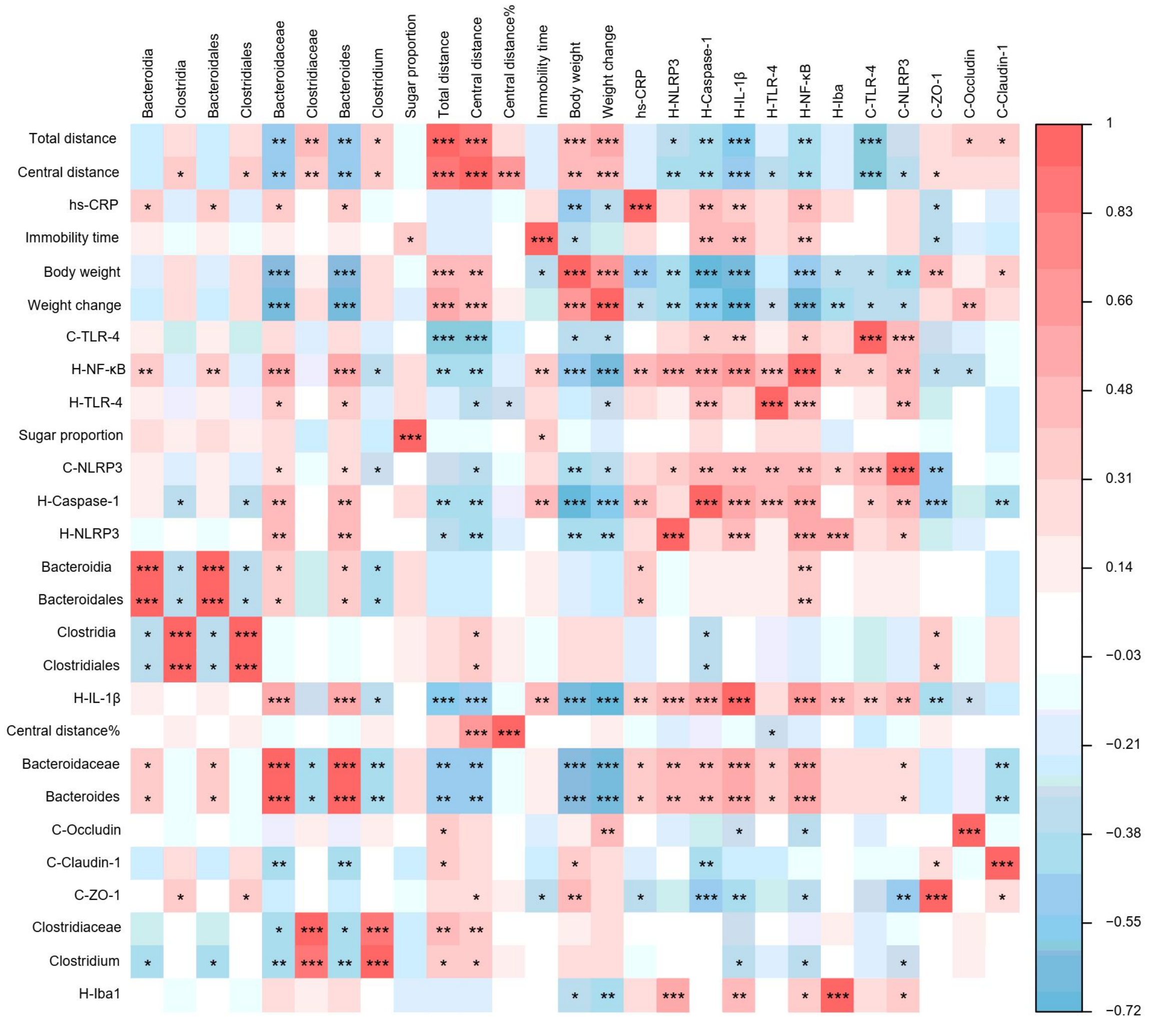

Fig-S3. The correlation among altered gut microbiota, inflammatory markers, permeability markers and depression-like behaviors. The spearman correlation analysis showed Bacteroidaceae, Bacteroides were positively correlated with inflammatory markers and depression-like behaviors, and negatively correlated with permeability markers. However, Clostridia and Clostridiales were negatively correlated with inflammatory markers and depression-like behaviors, and positively correlated with permeability markers; The inflammatory markers were negatively correlated with permeability markers and positively correlated with depression-like behaviors; Further, Bacteroidaceae, Bacteroides are negatively correlated with Clostridiaceae, Clostridium. “H-” represent Hippocampus, “C-” represent Colon. Pearson correlation analyses were implemented with FDR correction. The red and blue of the table represent positive and negative correlations respectively. *P*-value is

marked as follows: ***: $P \leq 0.001$; **: $P \leq 0.01$; *: $P \leq 0.05$. Source data are provided as a Source Data File.

Antibody array

The following steps were conducted by RayBiotech (Guangzhou, China). The intestinal mucosal homogenates were analyzed with a glass slide-based antibody cytokine array including 80 proteins (RayBiotech, GSH-INF-3,GSH-GF-1). A 100 μL sample diluent was added to each well and incubated at room temperature for 30 min. Then, the buffer was replaced with another 100 μL of sample and incubated at room temperature for 2 h. The samples were discarded and washed 5 times (5 min each) with 150 μL of 1 × Wash Buffer I and 2 times (5 min each) with 150 μL of 1 × Wash Buffer II at room temperature with gentle shaking. After that, 80 μL of the detection antibody cocktail were added to each well and incubated at room temperature for 2 h. The slide was washed 5 times (5 min each) with 150 μL of 1 × Wash Buffer I and then 2 times with 150 μL of 1 × Wash Buffer II at room temperature with gentle shaking. 80 microliters of Cy3 equivalent dye-conjugated streptavidin was added to each well and incubated at room temperature for 1 h. After 5 washes (5 min each), the signal was visualized through a laser scanner. The data were then visualized by a heatmap diagram (www.metaboanalyst.ca).

Animals and Experimental Procedure

Male C57BL/6J mice （n=44) were obtained from the SPF (Beijing) Biotechnology Co.,Ltd. (six weeks of age; 4-5 per cage), License Number: SCXK（jing）2019-0010. Mice were maintained in a temperature-controlled (21-23 °C) environment with a 12/12-h light-dark cycle. Mice were given standard chow and autoclaved water ad libitum. We created humanized mouse models through antibiotic treatment and human fecal microbial transplantation (FMT) according to previous studies with some modifications[1,2]. The gut microbiome of mice was depleted by oral gavage of a cocktail of antibiotics and then re-constructed with gut microbiota from Inflammatory depression patients， Non-inflammatory depression patients and healthy controls

(HCs). Finally, probiotics (Clostridium butyricum) were used in mouse models of inflammatory depression. Animal experimental procedure timeline is shown in manuscript.

The depletion of mouse intestinal microbiota

After acclimatization for 3 days, mice were gavaged every 12 h with 1 mg/kg amphotericin-B for 3 d followed by an antibiotic cocktail described Reikvam, et al[2] consisting of 50 mg/kg vancomycin, 100 mg/kg neomycin, 100mg/kg metronidazole, and 1 mg/kg amphotericin-B for 7 d. Ampicillin was provided in drinking water (1 g/L) ad libitum.

FMT

Fecal samples collected from the recruited subjects were prepared for microbiota transplantation according to the methods described in a previous study[3]. Briefly, approximately one gram of feces (one spoon) was collected and transferred to a 15-ml centrifuge tube immediately after patients or healthy volunteers defecated. Stool sample was suspended and diluted with 7.5 ml ice-cold reduced sterile PBS. The homogenate was centrifuged at 800g for 5 min at 4°C. 600μl of supernatant fluid and equal volume of 40% (volume rate) glycerin-PBS liquid were transferred to 1.5-ml Eppendorf tubes and vortexed. The stool samples were finally diluted by approximately 15X and the microbe-containing samples were stored in 20% glycerin-PBS liquid. Microbiota pools were made from donor suspensions of Inflammatory depression patients， Non-inflammatory depression patients and HCs and held at -80 °C until transplantation. Forty-eight hours after the final gavage of antibiotics, mice were randomly classified into three groups, and received oral gavage of the microbiota suspension (10 ul/g body weight) for 3 weeks during 8:00 a.m. to 9:00 a.m (The first week: Continuous gavage from Monday to Friday, once a day; The second and third week: Monday, Wednesday and Friday, every other day) to reconstruct gut microbiota.

Behavioral testing

All behavioral tests were conducted 24h after fecal transplantation and probiotic intervention. Animals were given 30 mins of habituation in the behavioral testing room before testing began in order to minimize novelty or stress effects. All behavioral tests were performed in a quiet, soundproof room with low light and the mice were returned to their cages after testing. Sucrose preference test (SPT)， Open-field test（OFT）and tail suspension test (TST) were used to evaluate anhedonia, anxiety and despair of mice respectively as described before. These results were either analyzed using a video computerized tracking system or scored by manual observation.

SPT

This experiment is mainly used to evaluate the anhedonia of mice. Sucrose preference was tested using a sucrose solution and tap water two-bottle free-choice method[4]: Mice were presented with two bottles containing 1% sucrose solution or tap water for 1 h during the dark phase in the home cage after 20 h water deprivation, and intake volume was measured. Sucrose preference was calculated as the percentage of sucrose solution ingestion relative to the total amount of liquid consumption.

OFT

The experiment was used to evaluated the anxiety-like behavior of mice. Mice were placed individually in the corner of an open-field box (L × W × H, 100 cm ×100 cm ×40 cm) and allowed to explore freely for 6 min. Their spontaneous activities over the last 5 min were recorded. The total move distance was designated as an index of locomotor activity, while increased proportion of time or distance spent in the center (inner 25% of the surface area, away from the walls) indexes decreased anxiety[5].

TST

This experiment was used to evaluated the mice's desperate behavior[6]. After SPT and OFT, TST was performed. each animal was hanged individually by adhesive tape separated by opaque fences. The tape was placed 1 cm from the tip of their tail. The behaviors of animals were monitored for a period of 6 minutes. Animals are considered immobile when they stay motionless for 6 seconds, and their immobility

time was recorded.

Collection and detection of mice tissue and feces

After the behavioral test, mice were anesthetized with 10% chloral hydrate (0.1mL /10g) and blood was obtained after the eyeball is removed. These blood samples were centrifuged at 3000 rpm for 20 min to collect the serum samples, which were immediately frozen at -80 °C for biochemical assays, ELISA was used to measure the concentrations of hs-CRP according to the manufacturer’s instructions.

Stool samples were collected from colon contents. 3-5 fecal pellets were collected from each mouse and stored in sterilization centrifuge tube at -80 °C and microbiota composition was interrogated using 16S rRNA sequencing.

The colon tissues were dissected from mice intestines. Out of two mucosal biopsy samples, one sample was placed in 10% formalin to perform Immunohistochemistry to measure the permeability biomakers such as Claudin-1、ZO-1、Occludin. The remaining sigmoid biopsies were snapped frozen at -80°C until use and the expression of TLR-4, NF-κB, NLRP3, Caspase-1 were detected by quantitative polymerase chain reaction (qPCR).

Hippocampus was dissected from mice brain. Part of the sample was placed in 10% formalin to perform Immunofluorescence to measure the iba-1 to assess the amount and morphology of microglia. The remaining biopsies were snapped frozen at -80°C and the expression of TLR-4, NF-κB, NLRP3, Caspase-1 were detected by qPCR.

Fecal sample DNA extraction, 16S rRNA gene sequencing, and bioinformatics data analysis

In order to collect fecal DNA, we implemented the QIAamp® DNA Stool Mini Kit (Qiagen, Hilden, Germany), following the maker’s instructions. By utilizing a NanoDrop One spectrophotometer (Thermo Fisher Scientific, Fitchburg, WI), we quantified the DNA, and measured the integrity and size using 0.8% agarose gel electrophoresis. In order to amplify the 16S rRNA gene of bacteria, we utilized isolated DNA following the 338F universal primers (5′-ACTCCTACGGG

AGGCAGCA-3′) and 806R (5′-GGACTACHVGGGTWTCTAAT-3′), focusing on the hypervariable regions V3-V4 of bacterial 16S rRNA. The illumina Novaseq PE250 platform by Personal Biotechnology, Co., Ltd. was used to conduct PCR amplification, preparation of sequencing library (Shanghai, China).

The open-source software QIIME 2 (version: 2019.1) was used to process the 16S rRNA raw sequencing data[7]. We demultiplexed the sequences and removed the V3/V4 primers with cutadapt (v2.8). We also implemented the DADA2 plugin package for the amplicon workflow: quality filtering, sequence truncation, denoise (error correction), sample inference, merging of paired-end reads, chimera identification and removal, singletons removal, and dereplication of sequences into amplicon sequence variants (ASV) with 100% sequence similarity[8]. Any OTUs found to have a frequency of < 0.1% of the total number of reads were excluded. a Naive-Bayes classifier trained against the SILVA 132 database[9] was used to perform taxonomic classification, targeting the V3/V4 region of the 16S rRNA. Using QIIME 2 (version: 2019.1), we calculated the alpha and beta diversity metrics. Alpha diversity indices of the Abundance-based Shannon index, Simpson index, Observed Species index, Chao index, Faith’s PD index, Pielou’s evenness index, and Good’s coverage index were taken based on rarefied sequence count. The permanova analysis based on Jaccard dissimilarity and an unsupervised principal coordinates analysis (PCoA) of Jaccard dissimilarity was used to conduct beta diversity analysis in order to identify variances of microbiome composition profiles at the ASV level. Linear discriminant analysis effect size (LEfSe) analysis[10] was implemented to locate distinguishing taxa among groups at genus level, and to conceptualize the results in a cladogram and bar plot. We used Spearman’s correlation coefficient to create the correlation matrix among microbiome composition, cytokines and HAMD. The online genescloud platform (https://www.genescloud.cn/chart /CorHeatmap) was used to generate a heat map of the correlation matrix. To find which gut microbiota could act as biomarkers for discriminating between MDD patients and HCs at the genus/ASV level, we applied a Random Forest (RF) and Boruta machine learning algorithm[11], and the region below the receiver operating characteristic curve (AUC) was utilized to measure the

classification performance.

Metagenome shotgun sequencing

Total microbial genomic DNA samples were extracted using the DNeasyPowerSoil Kit (QIAGEN, Inc., Netherlands), following the manufacturer's instructions. The quantity and quality of extracted DNAs were measured using a NanoDrop ND-1000 spectrophotometer (Thermo Fisher Scientific, Waltham, MA,USA) and agarose gel electrophoresis, respectively. The extracted microbial DNA was processed to construct metagenome shotgun sequencing libraries with insert sizes of 400 bp by using the Illumina TruSeq Nano DNA LT Library Preparation Kit. Each library was sequenced by the Illumina HiSeq X-ten platform (Illumina, USA) with PE150 strategy at Personal Biotechnology Co., Ltd. (Shanghai, China).

Raw sequencing reads were processed to obtain quality-filtered reads for further analysis. The sequencing adapters were removed from sequencing reads using Cutadapt (v1.2.1). Low quality reads were trimmed by using a sliding-window algorithm. The sequencing reads were aligned to the host genome using BWA to remove host contamination. Once quality-filtered reads were obtained, they were de novo assembled to construct the metagenome for each sample by IDBA-UD (Iterative De Bruijn graph Assembler for sequencing data with highly Uneven Depth). All coding regions (CDS) of metagenomic scaffolds longer than 300 bp were predicted by MetaGeneMark. CDS sequences of all samples were clustered by CD-HIT at 90% protein sequence identity, to obtain a non-redundant gene catalog. Gene abundance in each sample was estimated by soap.coverage based on the number of aligned reads. The lowest common ancestor taxonomy of the non-redundant genes was obtained by aligning them against the NCBI-NT database by BLASTN (e value < 0.001). Similarly, the functional profiles of the non-redundant genes were obtained by annotation against the KEGG and EggNOG databases using the DIAMOND alignment algorithm at Shanghai Personal Biotechnology Co., Ltd (Shanghai, China).

Targeted metabolomics analysis of SCFAs in plasma

Targeted SCFA panel profiling was performed by Metabolon in plasma samples from participants. 7 SCFAs: acetic acid (C2), propionic acid (C3), isobutyric acid (C4),

butyric acid (C4), isovaleric acid (C5), valeric acid (C5) and caproic acid (hexanoic acid, C6) were quantified by gas chromatography-mass spectrometry (GC-MS). Samples were extracted in 50 μL of 15% phosphoric acid with 10 μL of 75 μg/mL 4-methylvaleric acid solution as IS and 140 μL ether. Subsequently, the samples were centrifuged at 4 °C for 10 min at 12000 rpm after vortexing for 1 min and the supernatant was transferred into the vial prior to GC- MS analysis. immediately prior to each run. LC-MS/MS raw data were collected and processed using AB SCIEX software Analyst 1.6.2. Data reduction was performed using Microsoft Excel 2020. The GC analysis was performed on trace 1300 gas chromatograph (Thermo Fisher Scientific, USA). The GC was fitted with a capillary column Agilent HP- INNOWAX (30 m × 0.25 mm ID × 0.25 μm) and helium was used as the carrier gas at 1 mL/min. Injection was made in split mode at 10:1 with an injection volume of 1 μL and an injector temperature of 250 °C. The temperature of the ion source and interface were 300 °C and 250 °C, respectively. The column temperature was programmed to increase from an initial temperature of 90°C, followed by an increase to 120 °C at 10°C/min, and to 150 °C at 5 °C/min, and finally to 250 °C at 25 °C/min which was maintained for 2 min (total run-time of 15 min). Mass spectrometric detection of metabolites was performed on ISQ 7000 (Thermo Fisher Scientific, USA) with electron impact ionization mode. Single ion monitoring (SIM) mode was used with the electron energy of 70 eV. MS Convert software (Proteowizard, v3.0.8789) was applied to convert ".raw" format raw data to ".mzML" format, which would be used for downstream analysis. Extracted-ion chromatograms for each ion were abstracted based on the information of diagnostic ions and quantification ions. Peak areas would be detected by retention times for each compound. The calibration curves were obtained as plots of the peak area ratio of the target compounds to an internal standard versus the target compound concentration. The peak area ratio of target compounds to an internal standard was put into the formula in 2.2.2 to calculate the concentration in samples. In calculation, all metabolites concentrations lesser than 0 were reported as not detected (ND). To assess the technical precision of each

experiment, the relative standard deviation of peak areas was calculated for every compound detected in the QC sample (RSD = 100 * standard deviation / average of peak areas) with ideal RSD < 15%.

qPCR analysis

Hippocampus and colon tissues were isolated from the experimental mice and the expression of TLR-4, NF-κB, NLRP3, Caspase-1 were detected by qPCR as described in a previous study[12]. Briefly, and homogenized in TRIzol Reagent (Invitrogen), and total RNA was isolated according to the manufacturer's instructions. RNA (1 μg) was reverse transcribed into complementary DNA using HiScript II Select qRT SuperMix (Vazyme, Nanjing, China). Quantitative polymerase chain reaction was performed using gene-specific primer sets and SYBR Green (Vazyme, Nanjing, China) on a real-time PCR detection system (Bio-Rad). The primer sequences used for amplification were as follows:

mNLRP3 F (5′-ATTACCCGCCCGAGAAAGG-3′),

mNLRP3 R (5′-ATTACCCGCCCGAGAAAGG-3′),

mCaspase-1 F (5′-ACAAGGCACGGGACCTATG-3′),

mCaspase-1R (5′-TCCCAGTCAGTCCTGGAAATG-3′),

mTLR-4 F (5′-TGGTTGCAGAAAATGCCAGG-3′),

mTLR-4 R (5′-TCATCAGGGACTTTGCTGAGTT-3′),

mNF-KB F (5′-AGTCCATTGCATCGTCCCAA-3′),

mNF-KB R (5′-TCTGTGCGTGGCAACTACAT-3′),

IL-1β F (5′-GAAATGCCACCTTTTGACAGTG-3′),

IL-1β R (5′-TGGATGCTCTCATCAGGACAG-3′),

All the primers were provided by GENEray Biotechnology (Shanghai, China). The reaction conditions were 94 °C for 30 s followed by 40 cycles of 95 °C for 5 s and 60 °C for 30 s. Relative expression was calculated using the 2^-△△ct method with β-actin serving as the reference housekeeping gene. The expression of each target gene was normalized to β-actin expression, and the normalized data were presented as the fold change in gene expression in treated mice compared with control mice.

Intestinal mucosa immunohistochemistry analysis

1. Deparaffinzing and rehydrating the paraffin section: put the sections into xylene I for 15 minutes--xylene II for 15 minutes--xylene III for 15 minutes--absolute ethanol I for 5 minutes--absolute ethanol II for 5 minutes--85% alcohol for 5 minutes--75% alcohol for 5 minutes--rinse in distilled water.

2. Antigen retrieval: The intestinal mucosa sections are placed in a repair box filled with citric acid(PH6.0) antigen retrieval buffer for antigen retrieval in a microwave oven, heated on medium power for 8 minutes until boiling, then turned off the microwave oven, kept warm for 8 minutes and then transferred to medium-low power for heating 7minutes. During this process, excessive evaporation of buffer should be prevented and the sections should not be allowed to dry. To cool to room temperature before proceeding, the sections are placed in PBS(PH7.4) and shaken on the decolorization shaker 3 times for 5 minutes each.

3. Blocking endogenous peroxidase activity: the sections are placed in 3% hydrogen peroxide and incubated at room temperature in darkness for 25 minutes. The sections are placed in PBS(PH7.4) and shaken on a decolorizing shaper 3 times for 5 minutes each.

4. Serum sealing: 3%BSA was added to the circle to evenly cover the tissue, and the tissues are sealed for 30 minutes at room temperature. (Primary antibody is sealed with normal rabbit serum from goat source and other sources are sealed with BSA).

5. Primary antibody incubation: the sealing solution is gently removed, the primary antibody primary antibodies against Claudin-1 、ZO-1 、Occludin prepared with PBS(PH7.4) in a certain proportion is added to the sections, and the sections are placed flat in a wet box and incubated overnight at 4°C.

6. Secondary antibody incubation: the sections are placed in PBS(PH7.4) and washed by shaking on the decolorizing shaker 3 times for 5 minutes each. After the sections are slightly shaken and dried, the tissues are covered with secondary antibody (HRP labeled) from the corresponding species of primary antibody and incubated at room temperature for 50 minutes.

7. DAB chromogenic reaction: the sections are placed in PBS(PH7.4) and shaken on

the decoloring shaker 3 times for 5 minutes each. DAB color developing solution newly prepared is added in the circle after the sections are slightly dried. The color developing time is controlled under the microscope. The positive is brownish yellow.Rinse the sections with tap water to stop the reaction.

8. Nucleus counterstaining: the sections are counterstained with hematoxylin stain solution for about 3 minutes; washed with tap water; differentiated with hematoxylin differentiation solution for several seconds; washed with tap water; treated with hematoxylin returning blue solution; washed with running water.

9. Dehydration and mounting: place the section in 75% alcohol for 5 minutes--85% alcohol for 5 minutes--absolute ethanol I 5 minutes--anhydrous ethanol II 5 minutes--n-butanol 5 minutes--xylene I 5 minutes, dehydrated and transparent, remove the sections from xylene and let them dry slightly, then mount the sections with neutral gum.

10.Visualize staining of tissue under a microscope (Nikon, Japan) , acquisitive and analysis image.

11. Area density analysis：Eclipse CI-L photo microscope was used to select the target area of tissues for 200-fold imaging. During imaging, tissues should be filled with the whole field of vision as far as possible to ensure the consistency of background light in each photo.After imaging, image-Pro Plus 6.0 analysis software was used to uniformly measure the positive integral optical density (IDO)(Fig 1B) of the three fields and the corresponding tissue area（Fig 1D），then calculate the Areal density (IOD/ Area).

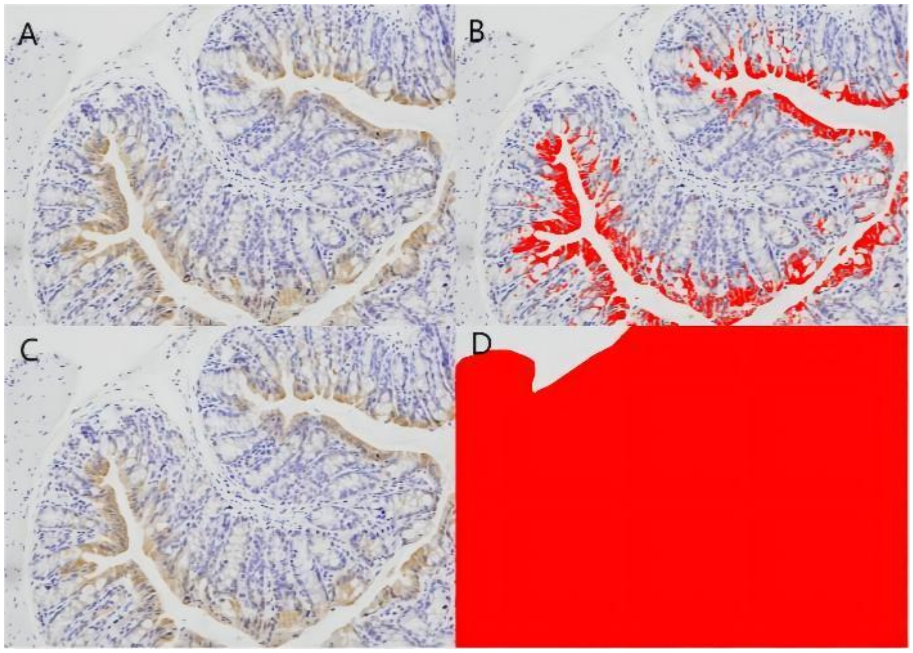

Fig-S4. Diagram of the application of the analysis software in immunohistochemistry analysis

Immunofluorescence staining analysis

1. Deparaffinize and rehydrate: incubate sections in 2 changes of xylene, 15 min each. Dehydrate in 2 changes of pure ethanol for 5 min, followed by dehydrate in gradient ethanol of 85% and 75% ethanol, respectively, 5 min each. Wash in distilled water.

2. Antigen retrieval: immerse the Hippocampus slides in EDTA antigen retrieval buffer (pH 8.0) and maintain at a sub-boiling temperature for 8 min, standing for 8 min and then followed by another sub-boiling temperature for 7 min. Be sure to prevent buffer solution evaporate. Let air cooling. Wash three times with PBS (pH 7.4) in a Rocker device, 5 min each. Use the right antigen retrieval buffer and heat extent according to tissue characteristics.

3. Circle and Serum blocking: eliminate obvious liquid, mark the objective tissue with liquid

blocker pen. Add 3% BSA to cover the marked tissue to block non-specific binding for 30 min .

Cover objective area with 10% donkey serum (for the case of primary antibody originated from

goat) or 3% BSA (for the case of primary antibody originated from others)

4. Primary antibody: throw away the blocking solution slightly. Incubate slides with primary

Antibody against Iba1 (diluted with PBS appropriately) overnight at 4 ℃, placed in a wet box containing a little water.

5. Secondary antibody: wash slides three times with PBS (pH 7.4) in a Rocker device, 5 min each.

Then throw away liquid slightly. Cover objective tissue with secondary antibody (appropriately respond to primary antibody in species), incubate at room temperature for 50 min in dark condition.

6. DAPI counterstain in nucleus: wash three times with PBS (pH 7.4) in a Rocker device, 5 min each. Then incubate with DAPI solution at room temperature for 10 min,

kept in dark place.

7. Spontaneous fluorescence quenching : wash three times with PBS (pH 7.4) in a Rocker device, 5 min each . Add spontaneous fluorescence quenching reagent to incubate for 5 min. Wash in running tap water for 10 min.

8. Mount: Throw away liquid slightly, then cover slip with anti-fade mounting medium.

9. Microscopy detection and collect images by Fluorescent Microscopy (Nikon, Japan) .

10. Area density analysis **:** Eclipse CI-L photo microscope was used to select the target area of tissues for 200-fold imaging. During imaging, tissues should be filled with the whole field of vision as far as possible to ensure the consistency of background light in each photo. After imaging, image-Pro Plus 6.0 analysis software was used to converted the green or red fluorescent monochrome photos to black and white (Fig 2B) and the same black was selected as the unified criterion for judging the positive value in all the photos. Then measure the positive integral optical density (IDO)(Fig 2B) from three fields and the corresponding tissue area (Fig 2D). Further, calculate the Areal density (IOD/ Area).

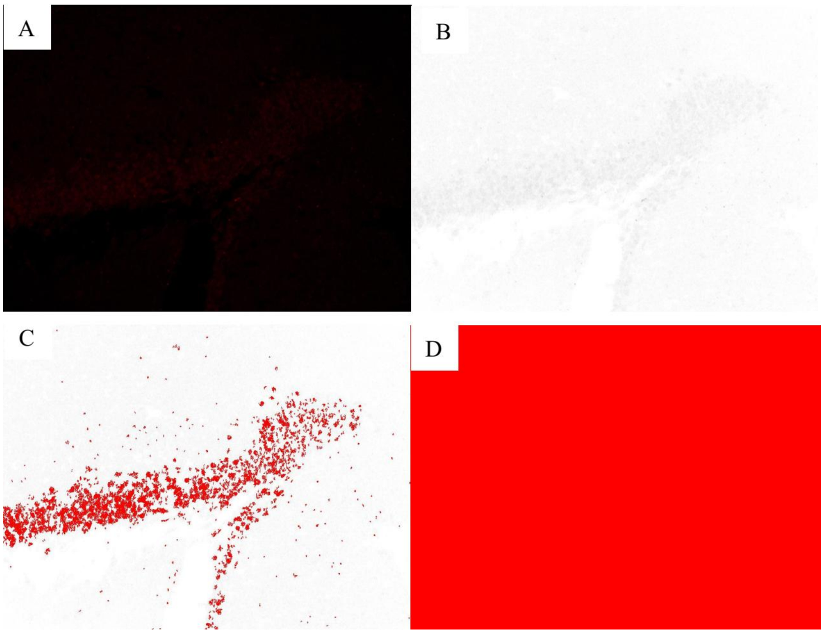

Fig-S5. Diagram of the application of the analysis software in immunofluorescence staining analysis.

Supplementary References

1. Bruce-Keller, A. J. et al. Obese-type gut microbiota induce neurobehavioral changes in the absence of obesity. *Biol. Psychiatry* **77**, 607-615 (2015).
2. Zhu, F. et al. Transplantation of microbiota from drug-free patients with schizophrenia causes schizophrenia-like abnormal behaviors and dysregulated kynurenine metabolism in mice. *Mol. Psychiatry* **25**, 2905-2918 (2020).
3. Hintze, K. J. et al. Broad scope method for creating humanized animal models for animal health and disease research through antibiotic treatment and human fecal transfer. *Gut Microbes* **5**, 183-191 (2014).
4. Willner, P., Towell, A., Sampson, D., Sophokleous, S. & Muscat, R. Reduction of sucrose preference by chronic unpredictable mild stress, and its restoration by a tricyclic antidepressant. *Psychopharmacology (Berl.)* **93**, 358-364 (1987).
5. Paré, W. P. Open field, learned helplessness, conditioned defensive burying, and forced-swim tests in WKY rats. *Physiol. Behav.* **55**, 433-439 (1994).
6. Cryan, J. F., Mombereau, C. & Vassout, A. The tail suspension test as a model for assessing antidepressant activity: review of pharmacological and genetic studies in mice. *Neurosci Biobehav Rev* **29**, 571-625 (2005).
7. Bolyen, E. et al. Author Correction: Reproducible, interactive, scalable and extensible microbiome data science using QIIME 2. *Nat. Biotechnol.* **37**, 1091 (2019).
8. Callahan, B. J., McMurdie, P. J., Rosen, M. J., Han, A. W., Johnson, A. J. & Holmes, S. P. DADA2: High-resolution sample inference from Illumina amplicon data. *Nat. Methods* **13**, 581-583 (2016).
9. Quast, C. et al. The SILVA ribosomal RNA gene database project: improved data processing and web-based tools. *Nucleic Acids Res.* **41**, D590-596 (2013).
10. Segata, N. et al. Metagenomic biomarker discovery and explanation. *Genome Biol.* **12**, R60 (2011).
11. Speiser, J. L., Miller, M. E., Tooze, J. & Ip, E. A Comparison of Random Forest Variable Selection Methods for Classification Prediction Modeling. *EXPERT SYSTEMS WITH APPLICATIONS* **134**, 93-101 (2019).
12. Li, J. M. et al. Dietary fructose-induced gut dysbiosis promotes mouse hippocampal neuroinflammation: a benefit of short-chain fatty acids. *Microbiome* **7**, 98 (2019).
